# A Metabolomic Study of the Analgesic Effect of Lappaconitine Hydrobromide (LAH) on Inflammatory Pain

**DOI:** 10.3390/metabo12100923

**Published:** 2022-09-29

**Authors:** Xu Li, Xueqi Wang, Zhengdou Li, Ying Mao, Zhao Liu, Xiaoxiao Liu, Xinliang Zhu, Ji Zhang

**Affiliations:** 1 College of Life Science, Northwest Normal University, Lanzhou 730070, China; 2 Institute of New Rural Development, Northwest Normal University, Lanzhou 730070, China; 3 Bioactive Products Engineering Research Center for Gansu Distinctive Plants, Lanzhou 730070, China; 4 Department of Orthopaedic Surgery, Orthopaedic Institute, The First Affiliated Hospital, Soochow University, Suzhou 215006, China; 5 Lanzhou Institute of Food and Drug Control, Lanzhou 740050, China

**Keywords:** inflammatory pain, complete Freund’s adjuvant (CFA), lappaconitine hydrobromide (LAH), dorsal root ganglion (DRG), metabolomics

## Abstract

Lappaconitine (LA) is a C-18 diterpene alkaloid isolated from *Aconitum sinomontanum* Nakai that has been shown to relieve mild to moderate discomfort. Various researchers have tried to explain the underlying mechanism of LA’s effects on chronic pain. This article uses metabolomics technology to investigate the metabolite alterations in the dorsal root ganglion (DRG) when lappaconitine hydrobromide (LAH) was injected in an inflammatory pain model, to explain the molecular mechanism of its analgesia from a metabolomics perspective. The pain model used in this study was a complete Freund’s adjuvant (CFA)-induced inflammatory pain model in rats. There were two treatment groups receiving different dosages of LAH (4 mg/kg LAH and 8 mg/kg LAH). The analgesic mechanism of LAH was investigated with an analgesic behavioral test, tissue sections, and metabolomics. The results of the analgesic behavioral experiment showed that both 4 mg/kg LAH and 8 mg/kg LAH could significantly improve the paw withdrawal latency (PWL) of rats. The tissue section results showed that LAH could reduce the inflammatory response and enlargement of the paw and ankle of rats and that there was no significant difference in the tissue sections of the DRG. The metabolomics results showed that retinol metabolism and glycerophospholipid metabolism in the CFA-induced inflammatory pain model were significantly affected and may exacerbate the inflammatory reactions and initiate persistent pain; in addition, the linoleic acid metabolism, arachidonic acid metabolism, and alanine, aspartate, and glutamate metabolism were also slightly affected. Among them, the alpha-linolenic acid metabolism was up-regulated after LAH treatment, while the retinol metabolism was down-regulated. These results suggest that LAH could effectively reduce inflammatory pain and might achieve this by regulating the lipid metabolism in the rat DRG.

## 1. Introduction

The International Pain Society (IASP) has recently issued a new definition of pain: “Pain is an unpleasant sensory and emotional experience related to actual or potential tissue damage or a similar experience” [1]. The perception of pain is different from nociceptive sensation. Nociceptive sensation refers to a type of neural activity of the nervous system in response to nociceptive stimuli, including physiological activity, biochemical response, and other cellular and molecular biological response processes that make up part of the meaning of the term “pain”. Pain is usually an adaptive and protective mechanism, but pain can also negatively impact physical function, mental health, and social life [1]. Pain has become a major problem that affects people’s lives and health, and hinders social productivity in China and around the world [2]. According to the National Health Interview Survey (NHIS) [3], 50.2 million adults in the US (20.5%) suffer from chronic pain, which significantly affects productivity and costs the economy USD 300 billion annually.

Lappaconitine (LA) is a C-18 diterpenoid alkaloid isolated from *Aconitum sinomontanum* Nakai, which is in the Ranunculaceae family [4]. It has obvious antipyretic effects, as well as analgesic, anti-inflammatory, and anti-swelling effects but no teratogenic mutagenic effects. Its analgesic effect is equivalent to pethidine [5], which is seven times more powerful than aminopyrine [6]. LA has been used clinically for many years because of its effective analgesic and non-addictive [7] properties [5,8]. The analgesic effect of LA has been widely demonstrated in previous research [9]. The use of an intraperitoneal injection or gavage of LA could effectively increase the pain threshold in a range of animals in numerous pain measurement tests, such as the hot plate, torsional response, photothermal radiation, tail shaking, and tail pressure tests. The pain response induced by acetic acid, formaldehyde, and hot plate tests was significantly inhibited and the paw edema induced by egg albumen in the rat and the ear edema induced by xylene in the mouse were all significantly suppressed by LA [9].

There is no uniformity in the molecular mechanism of analgesia by LA. The traditional view is that LA blocks voltage-gated sodium channels by binding to sodium channel site 2 and reducing the Na^+^ inward flow, thus further blocking K^+^ inward flow to influence the inhibition of action and potential generation and slowing the onset of pain [10]. LA inhibits voltage-gated sodium channels, promotes the release of norepinephrine, and inhibits the release of substance P in the synaptic cleft, thereby exerting analgesic effects [11]. Ou Shan et al. [12] found that LA involves the decrease in expression and sensitization of the P2X3 receptors of the rat DRG neurons, following chronic constriction injury (CCI). X Guo and also M Ono et al. [13,14] showed that reducing the concentration of central norepinephrine and damaging serotonergic neurons in the central nervous system inhibited the analgesic effect of LA. Sun Mingli et al. [15] showed that the analgesic mechanism of aconite alkaloids was to stimulate the spinal microglia to produce the endogenous polypeptide, dynorphin A.

In recent years, DRG stimulation has been increasingly reported for the alleviation of chronic pain; targeted DRG therapy for the neuromodulation of chronic pain has emerged as an important treatment option. DRG neurons are primary sensory neurons that express a variety of noxious receptors and ion channels [16]. DRG neurons are responsible for sensory transduction and modulation from the periphery; changes in the plasticity and modality of DRG neurons may be a hallmark of chronic pain [16,17]. Chronic rational pain includes neuropathic pain caused by nerve damage and inflammatory pain caused by tissue damage. Inflammation-related pain triggered by CFA has been extensively studied; it is released from the inflamed parts by directly acting on sensory receptors in the primary sensory neurons to transmit pain signals, or by activating receptors, G-proteins, and the second-messenger coupling that indirectly regulate pain signals to induce peripheral and central complex processing changes [18]. CFA-induced inflammation promotes the release of inflammatory mediators from cells, including cytokines (such as TNF-α, IL-1β, and IL-6) and chemokines (such as CCL2, CXCL1, and CX3CL1) [19,20]. Inducible nitric oxide synthase (iNOS) and prostaglandin E2 (PGE2) could increase neuronal excitability and synaptic transmission, leading to pain [21,22,23]. 

Metabolomics technology is used to qualitatively and quantitatively analyze all low molecular weight metabolites in the cells of a biological system at a specific time point and under specific conditions, to quantitatively describe the totality of endogenous metabolites and their response to changes in internal conditions and external causes [24]. Despite the rapid development, mature technology, and great potential of metabolomics technology, it has rarely been used in the field of pain [25,26]. Metabolite levels can reflect normal or altered metabolic pathways during pain onset, identify the biomarkers of pain onset [27,28], facilitate our exploration of the close association between characteristic metabolites and cellular as well as organismal phenotypes, and improve our systematic understanding of the mechanisms of pain onset; likewise, metabolomics can help identify new and more precise pathways to achieving therapeutic targets [29,30]. In this work, metabolomics techniques were used to study CFA-induced inflammatory pain and the changes in metabolite levels in the DRG of rats after LAH treatment, analyzing the metabolic pathways involved in the differential metabolites, thereby providing new insights to elucidate the analgesic mechanism of LAH treatment of inflammatory pain.

## 2. Materials and Methods

### 2.1. Animals and Samples 

**Figure 1:**
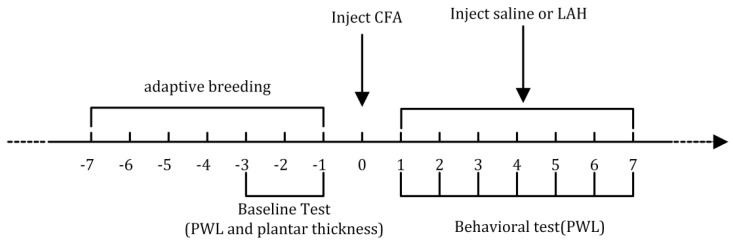
Animal experiment protocol.

SPF adult male Wistar rats (n = 24), 6~8 weeks old, and weight 200–220 g were provided by the Lanzhou Institute of Veterinary Research, Chinese Academy of Agricultural Sciences. Rats were fed an adaptive diet (standard diet for rats) at a temperature of 25 ± 2 °C, relative humidity of 55 ± 5%, in natural light, and with a free diet (standard diet for rats) for one week before the experiment. The animals were divided into four groups (each group had 6 rats): (a) saline, (b) CFA+ saline, (c) CFA + 4 mg/kg LAH, and (d) CFA + 8 mg/kg LAH. Each model group was injected with 100 μL of 100% CFA (Cat. No. F5581, Sigma-Aldrich, St. Louis, MO, USA) to create an inflammatory pain model. The saline and CFA + saline groups were injected intraperitoneally with the same volume of saline each day. The CFA + 4 mg/kg LAH and CFA + 8 mg/kg LAH groups were injected with LAH (Gansu Lanyao Pharmaceutical Co., Ltd., Lanzhou, Gansu, China) in the abdominal cavity every day and the PWL of rats was measured for 2 h with a thermal prick pain instrument (PL-200, Chengdu Technology & Market Co., Ltd., Chengdu, Sichuang, China) after daily administration for 7 consecutive days (Figure 1). The animal experiment protocol was approved by the animal ethics committee of Xi’an Jiaotong University’s School of Life Science and Technology (approval number: SCXK (Shaan) 2017-003).

### 2.2. Paw Withdrawal Latency

The PWL of the rats was measured using the PL-200 thermal prick pain instrument (intensity set to 45%, cut-off after 45 s) to generate a variable intensity radiation beam with a high spotlight, which was focused by an infrared filter to irradiate the bottom of the rat’s foot; the latency time (s) from the beginning of irradiation to the appearance of the rat’s response (lifting, contracting, and licking the foot) was taken as the pain threshold. Generally, the average value was taken as the PWL value after 3 consecutive measurements. The rats were trained with photothermal radiation stimulation for three days before the behavioral test, screening to remove overly sensitive or unresponsive rats; rats with no significant difference in PWL were selected for the formal behavioral test. To reduce errors, before the PWL test, the rats were placed in the behavioral box for 30 min to calm down, after which photothermal stimulation was performed at 10-minute intervals.

### 2.3. Hematoxylin and Eosin (H&E) Staining

After the 7-day behavioral testing, the DRG of segments L3–L5 of the ipsilateral lumbar spine of rats in each experimental group were harvested, fixed with 4% paraformaldehyde (P0099-100ml, Beyotime Biotechnology, Shanghai, China) for 48 h, and sectioned (RM2016,, Leica Company, Wetzlar, German) after being embedded in paraffin (JB-P5, Wuhan Junjie Electronics Co., Ltd., Wuhan, Hubei, China). The histological procedure includes:

(1) Dewaxing: Xylene (10023418, Sinopharm Chemical Reagent Co., Ltd., Shanghai, China) I for 20 min; Xylene II for 20 min; 100% ethanol (100092683, Sinopharm Chemical Reagent Co., Ltd., Shanghai, China) I for 5 min; 100% ethanol II for 5 min; 75% ethanol for 5 min; rinsing with tap water.
(2) Staining sections with hematoxylin solution for 3–5 min and rinsing with tap water. Then, we treat the section with hematoxylin differentiation solution and then rinse with tap water. We then treat the section with hematoxylin Scott’s Tap Water bluing and rinse with tap water (HE dye solution set, G1003, Wuhan Servicebio Technology Co., Ltd., Wuhan, Hubei, China).
(3) Treating with 85% ethanol for 5 min; 95% ethanol for 5 min. Finally, we stained the sections with eosin dye for 5 min.
(4) Dehydrating, as follows: 100% ethanol I for 5 min; 100% ethanol II for 5 min; 100% ethanol III for 5 min; Xylene I for 5 min; Xylene II for 5 min. Finally, we seal it with neutral gum (10004160, Sinopharm Chemical Reagent Co., Ltd., Shanghai, China).
(5) Observing with a microscope (NIKON ECLIPSE E100, Nikon-Essilor Co., Ltd., Tokyo, Japan) for the purposes of inspection, image acquisition (NIKON DS-U3, Nikon-Essilor Co., Ltd., Tokyo, Japan), and analysis. The nucleus is blue, while the cytoplasm is red.

### 2.4. Preparation of Sample for LC-MS/MS

The DRG of the lumbar spine at L3–L5, ipsilateral to the posterior paw of rats, was obtained in a pre-cooled mixture (acetonitrile (Cat. No. 271004, Sigma-Aldrich, St. Louis, MO, USA): methanol (Cat. No.34860, Sigma-Aldrich, St. Louis, MO, USA): water = 2:2:1, *v*/*v*)), vortexed and ultrasonically treated at low temperature for 30 min, incubated at −20 °C for 10 min, centrifuged at 4 °C for 20 min at 14,000 g. We vacuum-dried the supernatant before Liquid chromatography-Mass spectrometer/Mass spectrometer (LC-MS/MS) analysis and samples were kept at −80 °C. Each sample received 150 mL of acetonitrile aqueous solution (acetonitrile: water = 1:1 *v*/*v*). The mixture was then vortexed for 1 min before centrifugation at 14,000 g for 15 min at 4 °C. Supernatants were kept in centrifuge tubes and underwent vacuum drying until there was no liquid. Each test sample was combined equally to make a quality control (QC) sample. The analytical method for the QC sample was the same as for the test samples, and the QC sample was tested in every four test samples to check the instrument’s stability and performance.

### 2.5. LC-MS/MS Analysis

The sample was analyzed using a Thermo Scientific™ Vanquish™ Ultra-high performance liquid-chromatography (UHPLC) system (Thermo Fisher Scientific Inc., Waltham, MA, USA) as previously described. A C18 column (Thermo Hypersil GOLD™ C18, 100 mm × 2.1 mm, 1.8 μm, Thermo Fisher Scientific Inc., Waltham, MA, USA), coupled to a Q-Exactive (Thermo Fisher Scientific Inc., Waltham, MA, USA) mass spectrometer, was used for the separation of metabolites. The mobile phase consisted of 0.1% formic acid (Cat. No. F0507, Sigma-Aldrich, St. Louis, MO, USA) in water (A) for the positive mode, 5 mM ammonium acetate (Cat. No. 73594, Sigma-Aldrich, St. Louis, MO, USA) in 0.1% formic acid water (A) for the negative mode, as well as acetonitrile (B) under the following gradient conditions: 0 min–1 min, 1% B; 1 min–8 min, 1%~99% B; 8–10 min, 99% B; 10 min–10.1 min, 99%~1% B; 10.1–12 min, 1% B. The flow rate was 0.3 mL/min, the column temperature was 35 °C, and the sample injection volume was 4 μL. 

Mass spectrometric (MS) analysis was performed with a Q-Exactive mass spectrometer, which used electrospray ionization sources in the positive and negative ionization modes. The operating parameters were operated as follows: positive polarity; spray voltage 4.0 kV (positive ion mode) or −3.6 kV (negative ion mode); funnel radio frequency (RF) lens value at 50; a capillary temperature of 400 °C. The flow rates for sheath gas, auxiliary gas, and sweep gas were set to 45, 15, and 0, respectively. Except where otherwise noted, data-dependent acquisition (DDA) using the full Mass Spectrometry-data-dependent MS/MS analysis (MS-ddMS^2^) setup was used. Full MS resolution was set to 70,000, and the mass range was set to 100–1500. For MS^2^ spectra, the resolution was set to 17,500. The normalized collision energy was set to 20%, 40%, and 60%. Dynamic exclusion values were set to 5 s.

### 2.6. Data Analysis

We used Microsoft Excel (V2021, Microsoft Corp, Redmond, WA, USA) to organize the data, while GraphPad Prism (version 9.0, GraphPad Software, CA, USA) and Originpro (V2022.SR1, OriginLab, Northampton, MA, USA.) were used to analyze and map the rat data of PWL. A one-way analysis of variance (ANOVA), preceded by a Bonferroni test (95% confidence interval), was carried out when comparing three or more groups. MZmine (V3.2.3, obtained from the project website at: http://mzmine.sourceforge.net/.) [31] was used to analyze the LC-MS raw data, with orthogonal partial least-squares discriminant analysis (OPLS-DA) in the MetaboAnalyst 5.0 web server (http://www.metaboanalyst.ca, accessed on 24 March 2022) and SIMCA (V.14.0, Umetrics Inc., Umea, Sweden.) software. A VIP > 1, the fold change (FC) value (FC > 2 and FC < 0.5), and Student’s *t*-test (*p* < 0.05) were used to determine the differential metabolites. We used the human metabolome database (HMDB) (http://www.hmdb.ca/, accessed on 24 March 2022) for retrieval compounds, while the MetaboAnalyst 5.0 web server (https://www.metaboanalyst.ca/, accessed on 24 March 2022) and the Kyoto Encyclopedia of Genes and Genomes (KEGG) (https://www.genome.jp/kegg/, accessed on 19 April 2022) were used to analyze the pathways. R 4.1.1 was used to create the bubble map of the pathway enrichment analysis. 

## 3. Results

### 3.1. Paw Withdrawal Latency

**Figure 2:**
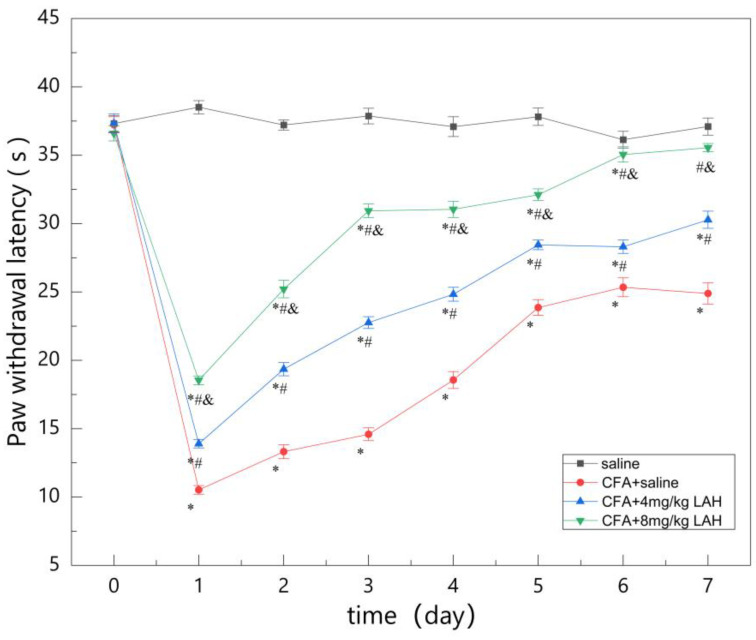
Paw withdrawal latency of rats over 7 days. *, compared to saline group, *p* < 0.05; #, compared to CFA + saline group, *p* < 0.05; &, compared to CFA + 4 mg/kg LAH group, *p* < 0.05.

The results of the 7-day test showed that CFA significantly reduced the PWL of rats, and both CFA + 4 mg/kg LAH and CFA + 8 mg/kg LAH increased the PWL of rats with CFA-induced inflammatory pain. In other words, LAH has a significant analgesic effect on CFA-induced inflammatory pain, while 8 mg/kg LAH has a more effective analgesic effect in a dose-dependent manner (Table 1, Figure 2).

### 3.2. H&E Staining

**Figure 3:**
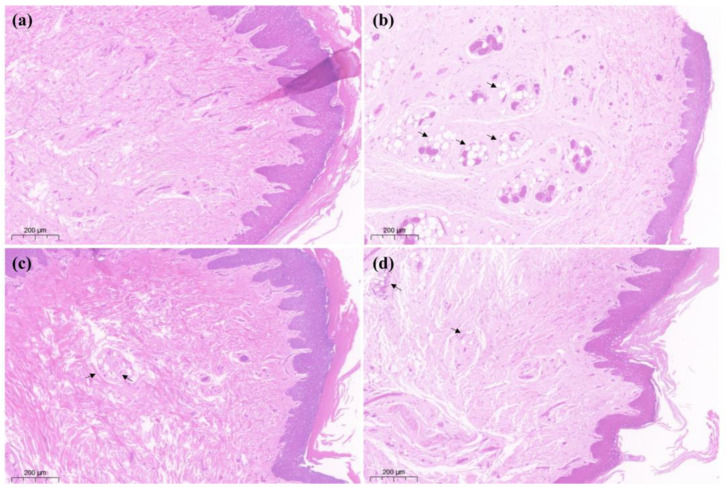
Hind soles H&E staining on day 7. (**a**) saline, (**b**) CFA + saline, (**c**) CFA + 4 mg/kg LAH, (**d**) CFA + 8 mg/kg LAH. Ruler: 200 μm. spongy and formed vesicles showed by “
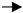
”.

The results of histopathological staining showed that the cells in the saline group (a) were densely arranged and had no inflammatory features. In the CFA + saline group (b), the tissue was spongy and formed vesicles (as shown by “
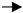
”), with a thin spiny layer and visible enlargement. In the treatment group CFA + 4 mg/kg LAH (c) and treatment group CFA + 8 mg/kg LAH (d), the small vesicles decreased, the spiny layer thickened, and the swelling reaction was alleviated. The results showed that the inflammatory swelling of the sole of the foot in rats could be significantly reduced by LAH (Figure 3).

**Figure 4:**
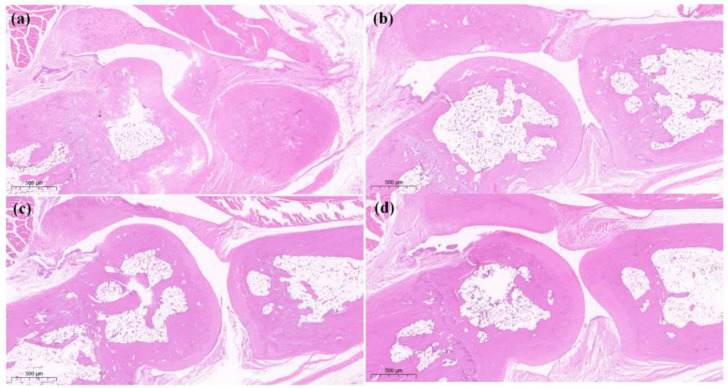
Posterior toe joint H&E staining on day 7. (**a**) saline, (**b**) CFA + saline, (**c**) CFA + 4 mg/kg LAH, (**d**) CFA + 8 mg/kg LAH. Ruler: 500 μm.

The results of the histopathological staining of the posterior paw and toe joints of rats showed that in the saline (a) group, the joint membrane tissue was smooth but not enlarged. The CFA + saline (b) group showed obvious enlargement of the joints and increased soft tissue swelling. In the treatment group, soft tissue enlargement was slightly reduced in treatment group (c) with CFA + 4 mg/kg LAH and treatment group (d) with CFA + 8 mg/kg LAH. The results showed that LAH could reduce the enlargement of the hind-foot ankles of rats (Figure 4).

**Figure 5:**
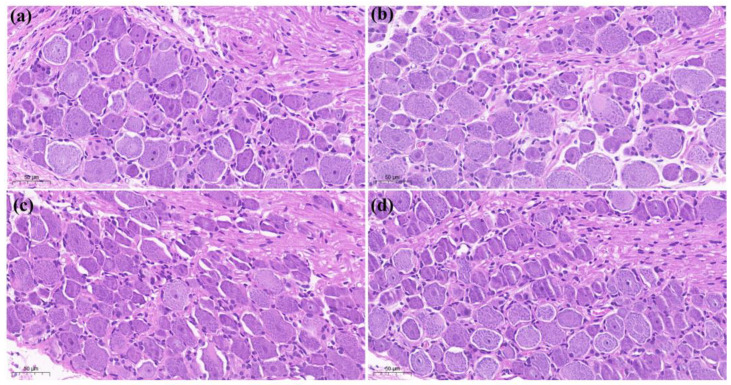
H&E staining of DRG (L5) on day 7: (**a**) saline, (**b**) CFA + saline, (**c**) CFA + 4 mg/kg LAH, and (**d**) CFA + 8 mg/kg LAH. Ruler: 50 μm.

Histopathological evaluation of DRG (L5) showed no significant difference between (a) saline, (b) CFA + saline, (c) CFA + 4 mg/kg LAH, and (d) CFA + 8 mg/kg LAH groups (Figure 5).

### 3.3. Differential Analysis of Metabolites

#### 3.3.1. Orthogonal Partial Least-Squares Discriminant Analysis

**Figure 6:**
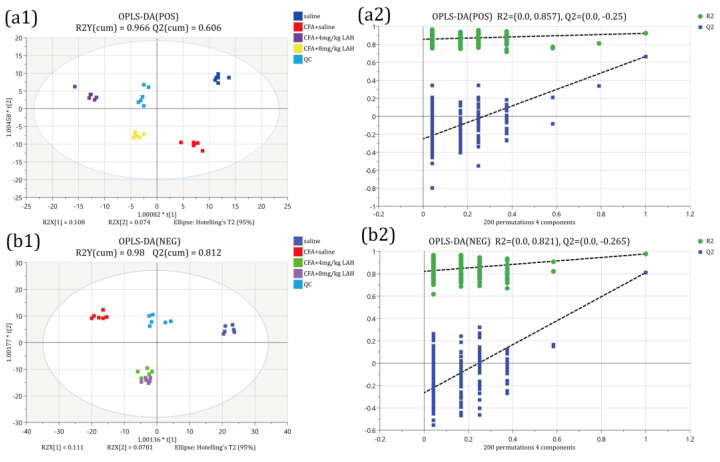
Orthogonal partial least-squares discriminant analysis (OPLS-DA). In positive (**a1**) and negative (**b1**) OPLS-DA score plots, each point represents one sample. The T-score axis represents the predictive variation among the classes, while the orthogonal T-score axis represents the variation orthogonal to the class-specific variation, and (**a2**,**b2**) is the overfitting analysis of the OPLS-DA model (two hundred permutations).

The OPLS-DA model was used to evaluate the differential metabolites; the OPLS-DA scores showed that other groups could be clearly separated from the CFA + saline group. The results of the OPLS-DA model overfitting analysis (200 hypothesis tests) showed that the model was of good quality and was not overfitted. At the same time, VIP was used to identify the main contributing metabolites of the OPLS-DA model. The QC samples were highly clustered and were located in the middle of each group, indicating that the experimental data were reliable and could be analyzed further (Figure 6).

#### 3.3.2. Univariate Analysis—Fold Change Analysis

**Figure 7:**
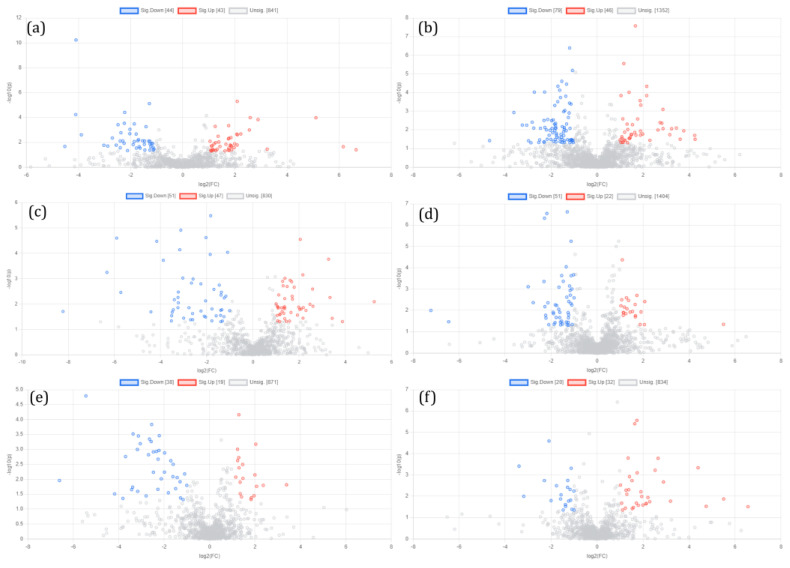
Univariate analysis-fold change analysis. Plots and different metabolite box-plots using MetaboAnalyst 5.0 (*p*-value < 0.05, fold change (FC) > 2 or FC < 0.5). Each point in the figure represents a compound; the blue dots represent the down-regulated differential metabolites, the red points represent the up-regulated differential metabolites: (**a**) CFA + saline vs. saline (positive ion mode), (**b**) CFA + saline vs. saline (negative ion mode), (**c**) CAF + 4 mg/kg LAH vs. CFA + saline (positive ion mode), (**d**) CAF + 4 mg/kg LAH vs. CFA + saline (negative ion mode), (**e**) CAF + 8 mg/kg LAH vs. CFA + saline (positive ion mode), and (**f**) CAF + 8 mg/kg LAH vs. CFA + saline (negative ion mode).

We set “*p*-value < 0.05 and fold change (FC) value (FC > 2 and FC < 0.5)” as the screening criteria; the results showed that 43 up-regulated differential metabolites were screened in the positive ion mode, with 44 down-regulated differential metabolites (a). In total, 46 up-regulated differential metabolites and 79 down-regulated differential metabolites were screened in the negative ion mode (b) in the CFA + saline vs. the saline group. In the case of CAF + 4 mg/kg LAH vs. the CFA + saline group, 47 up-regulated and 51 down-regulated differential metabolites were screened in positive ion mode (c), and 22 up-regulated and 51 down-regulated differential metabolites were screened in the negative ion mode (d). In CAF + 8 mg/kg LAH vs. CFA + saline group, 19 up-regulated and 38 down-regulated differential metabolites were screened in positive ion mode (e), and 32 up-regulated and 20 down-regulated differential metabolites were screened in negative ion mode (f) (Figure 7). The differential metabolites are given in the Supplementary Materials.

### 3.4. Differential Metabolic Pathway Enrichment Analysis

**Figure 8:**
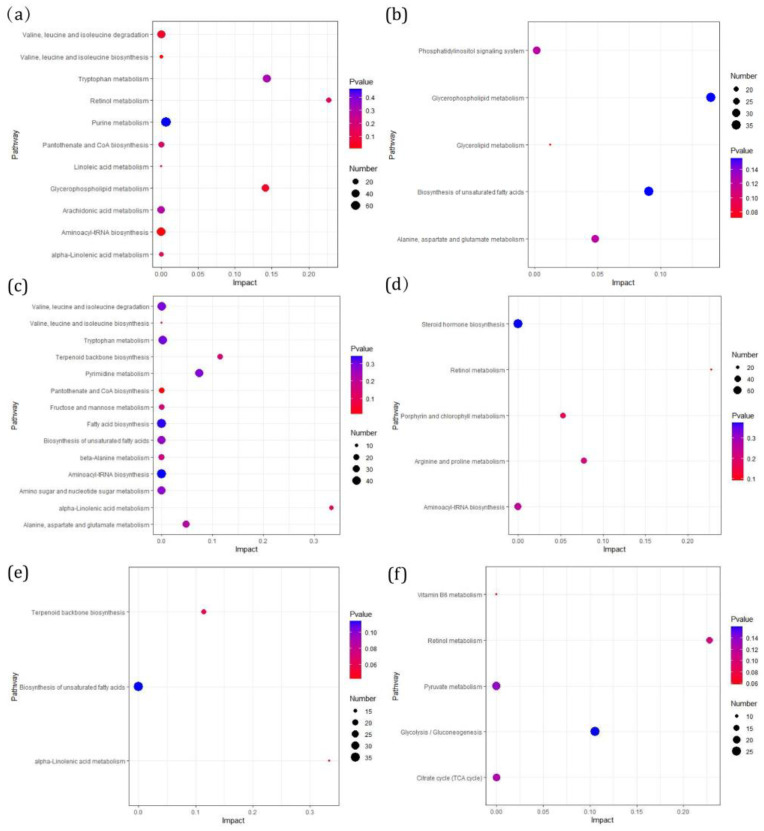
Differential metabolic pathway enrichment analysis. The names of the up-regulated metabolite enrichment pathways and down-regulated metabolite enrichment pathways are on the left-hand side. The horizontal axis is the enrichment factor (Impact), the bubble size shows the number of distinct metabolites engaged in this pathway, and the bubble color is the metabolic pathway’s super metric test (*p*-value). (**a**) CFA + saline vs. saline (up-regulated), (**b**) CFA + saline vs. saline (down-regulated), (**c**) CFA + 4 mg/kg LAH vs. CFA + saline (up-regulated), (**d**) CFA + 4 mg/kg LAH vs. CFA + saline (down-regulated), (**e**) CFA + 8 mg/kg LAH vs. CFA + saline (up-regulated), (**f**) CFA + 8 mg/kg LAH vs. CFA + saline (down-regulated).

Through further enrichment analysis of metabolic pathways, the results showed the metabolic pathways in the CFA-induced inflammatory pain group and the LAH treatment group. In the CFA + saline vs. saline group, the main up-regulated metabolic pathways were “Glycerophospholipid metabolism”, “Retinol metabolism”, “Linoleic acid metabolism”, and “Arachidonic acid metabolism” (a). The main down-regulated metabolic pathway was “Alanine, aspartate and glutamate metabolism” (b). In the CFA + 4 mg/kg LAH group vs. the CFA + saline group, the main up-regulated metabolic pathways were “Terpenoid backbone biosynthesis” and “Alpha-linolenic acid metabolism” (c); the main down-regulated metabolic pathway was “Retinol metabolism” (d). In the CFA + 8 mg/kg LAH vs. CFA + saline group, “Alpha-linolenic acid metabolism” and “Terpenoid backbone biosynthesis” were the main up-regulated metabolic pathways (e), and “Retinol metabolism” was the main down-regulated metabolic pathway (f) (Figure 8). The metabolic pathways are given in the Supplementary Materials. 

## 4. Discussion 

In this study, it was found that CFA-induced inflammatory and pain responses were significantly inhibited by LAH. Firstly, both low (4 mg/kg) and high (8 mg/kg) doses of LAH significantly increased the PWL values of rats, which indicated that LAH had good analgesic effects in the prevention of CFA-induced inflammatory pain. Secondly, the results of pathological tissue sections showed that LAH significantly reduced the inflammatory response of rat foot pads and alleviated the swelling symptoms of rat foot joints, which indicated that LAH also had anti-inflammatory and anti-swelling effects; the above results were consistent with the views expressed in the previous research [5,6,7,8,9]. Unfortunately, we did not see significant differences from the pathological tissue section results of DRG, which may require a more refined approach to distinguish subtle changes at the subcellular level.

The metabolomics results showed that the major upregulated metabolic pathways in the DRG of rats with CFA-induced inflammatory pain were the retinol metabolism and glycerophospholipid metabolism. The linoleic acid metabolism and arachidonic acid metabolism were also affected; the major downregulated metabolic pathways were alanine, aspartate, and glutamate metabolism. Polyunsaturated fatty acids (PUFAs) and arachidonic acids (AA) have been considered to be bioactive lipid molecules in glycerophospholipid metabolism and play an important role in proinflammatory signal transduction [32]. Some glycerophospholipids are activated and then converted into PUFAs to produce oxidized lipids and promote inflammatory pain [33]. For example, PG2, a product of cyclooxygenase (COX) in the arachidonic acid metabolism, can bind to EP receptors in neurons, activate protein kinases [34], and sensitize the transient receptor potential cation channel subfamily V member 1 (TRPV1), causing inflammatory pain [35]. The endogenous 9- and 13-hydroxyoctadecadienoic acid (HODE) promoted inflammation and activates the thermal reactivity of TRPV1 to produce pain [36]. The enzyme cytochrome P450 (CYP450) converts AA to epoxide eicosatrienoic acids (EETs); these EETs at certain concentrations or times promote persistent pain [37,38]. Our results suggest that the CFA-induced inflammatory pain model affects the lipid metabolism in the areas of glycerophospholipid metabolism, linoleic acid metabolism, and arachidonic acid metabolism. Proinflammatory lipids, such as AA, PG2, 9-hydroxyoctadecadienoic acid (9-HODE), or 13-hydroxyoctadecadienoic acid (13-HODE) are produced, which promote the expression and release of inflammatory cytokines and chemokines and cause inflammatory pain. 

Upon LAH treatment, the major upregulated metabolic pathway was the alpha-linolenic acid metabolism, while the major downregulated metabolic pathway was the retinol metabolism. The relationship between polyunsaturated fatty acids and pain has attracted much attention as one of the regulatory factors of pain. Studies have shown that n-3 fatty acids can reduce the pain caused by inflammation and neuropathy [39], while ω-3 polyunsaturated fatty acids present an attractive adjunct treatment for rheumatoid arthritis, inflammatory bowel disease, and joint pain [40]. Some studies have shown that TRPV1 is a new target for omega-3 polyunsaturated fatty acids [41]. Eicosapentaenoic acid (EPA) omega-3 fatty acid, an active metabolite in the alpha-linolenic acid (ALA) pathway, can effectively attenuate capsaicin-induced pain behavior in mice [42]. The novel mediator neuroprotectin D1 (NPD1) is biosynthesized from the ω-3 fatty acid docosahexaenoic acid (DHA) [43]. NPD1 effectively inhibits the capsaicin-induced TRPV1 current (IC50 = 0.4 nm), which is dissociated in neurons of the DRG [44]. With regard to inflammation, N-3-PUFA has a number of anti-inflammatory effects, including reducing the expression of adhesion molecules and adhesion interaction between leukocytes and endothelial cells, reducing the chemotactic response of leukocytes, and reducing the production of the classic inflammatory cytokines, TNFα, IL-1β, and IL-6 [45]. α-Linolenic acid is a regulator of arachidonic acid biosynthesis. Not only does it compete as a substrate for cyclooxygenase (EPA) and competes with arachidonic acid for the synthesis of prostaglandins and leukotrienes at the level of cyclooxygenase and lipoxygenase [46]), but n-3 fatty acids can also directly inhibit the activity of this enzyme (by inhibiting the activities of δ-6- and δ-5-desaturases) and can, thus, reduce arachidonic acid synthesis. These results suggest that LAH may exert anti-inflammatory and analgesic effects by regulating the levels of ω-3 and ω-6 polyunsaturated fatty acids.

Retinoids are structurally related derivatives of vitamin A (retinol) and the retinoid X receptor (RXR) for its active metabolite, retinoic acid (RA); they are activated only by 9-cis-retinoic acid (9-cis-RA) [47] and can act as fatty acid receptors in vivo [48]. Studies have shown that vitamin A plays a role in regulating glucose and fatty acid metabolism. RA regulates the expression level of the enzymes involved in fatty acid synthesis [49], while fatty acids and retinoids control the lipid metabolism by activating the peroxisome proliferator-activated receptor (PPAR)-RXR dimer [50]. Shijin Yin et al. [51] found that high retinoid concentrations trigger sensory hypersensitivity through the activation of TRPV1 receptors, while oral or intrathecal administration of all-trans retinoic acid (ATRA) can induce nociceptive behaviors in rodents [52]. In one study, pain was found to be induced by a plantar injection of LE135 [4-(7,8,9,10-tetrahydro-5,7,7,10,10-pentamethyl-5*H*-benzo[e]naphtho[2,3-b], a selective antagonist of the retinoic acid receptor beta (RAR β) and a potent activator of TRPV1 and TRPA1 receptors [53]. In terms of inflammation, RXR is an important regulator of macrophages and is a key player in inflammation and metabolic disorders. The arachidonic acid and RXR-α ligand-binding domains play important roles in the regulation of inflammation and innate immune response. In mice lacking RXRα in myeloid cells, leukocyte recruitment to the sites of inflammation is impaired [54]. Additionally, 9-cis-RA can reduce LPS-induced inflammation [55]. Both 9-cisRA and ATRA can stimulate the activation of RAR [56]. RXR also forms heterodimeric peroxisome proliferator-activated receptors (PPARs) with several other nuclear receptors [57], which are lipid-activated transcription factors. This plays an important role in inhibiting the expression of inflammatory genes [58]; both natural and synthetic peroxisome proliferator-activated receptor gamma (PPAR γ) agonists block the production of the proinflammatory cytokines TNFα, IL-6, and IL-1β in cultured monocytes [59].

This study provided evidence in terms of behavioral and pathological, as well as metabolomic responses during the treatment of inflammatory pain in rats by LAH. Further studies are certainly needed to explore and validate the specific mechanisms of the analgesic action of LAH. This will require the evaluation of key genes and proteins in the metabolic pathways that may be involved, to explain the scenarios in which LAH exerts an analgesic function by perturbing the metabolism network in DRG.

## 5. Conclusions

(1) Lappaconitine has a preferential analgesic effect on CFA-induced inflammatory pain behavior.
(2) CFA-induced inflammatory pain mainly triggered changes in the pathways of retinol metabolism, glycerophospholipid metabolism, and alanine, aspartate, and glutamate metabolism in the DRG of rats; the alpha-linolenic acid metabolism and retinol metabolism were significantly altered after LAH treatment.
(3) These results suggest that LAH could effectively reduce inflammatory pain and might achieve this through regulating the lipid metabolism in rat DRG.

## Figures and Tables

**Table 1:** Paw withdrawal latency of rats over 7 days.

Group	Saline	CFA + Saline	CFA + 4 mg/kg LAH	CFA + 8 mg/kg LAH
Baseline	37.31 ± 0.59	37.22 ± 0.59	37.33 ± 0.68	36.59 ± 0.55
Day 1	38.51 ± 0.48	10.52 ± 0.32 *	13.90 ± 0.31 *#	18.53 ± 0.32 *#&
Day 2	37.21 ± 0.37	13.32 ± 0.51 *	19.35 ± 0.49 *#	25.21 ± 0.64 *#&
Day 3	37.87 ± 0.57	14.59 ± 0.47 *	22.76 ± 0.43 *#	30.93 ± 0.54 *#&
Day 4	37.46 ± 0.72	18.56 ± 0.62 *	24.83 ± 0.51 *#	31.04 ± 0.59 *#&
Day 5	38.04 ± 0.63	23.86 ± 0.58 *	28.44 ± 0.35 *#	32.11 ± 0.43 *#&
Day 6	37.69 ± 0.61	25.34 ± 0.69 *	28.31 ± 0.49 *#	35.06 ± 0.56 *#&
Day 7	37.37 ± 0.63	24.88 ± 0.77 *	30.28 ± 0.63 *#	35.55 ± 0.30 #&

Means ± standard error (n = 6). *, compared to saline group, *p* < 0.05; #, compared to CFA + saline group, *p* < 0.05; &, compared to CFA + 4 mg/kg LAH group, *p* < 0.05.

## Data Availability

The data presented in this study are available in the article.

## Supplementary Materials

The following supporting information can be downloaded at: https://www.mdpi.com/article/10.3390/metabo12100923/s1, Table S1: Up-regulated differential metabolites (CFA + saline VS saline); Table S2: Down-regulated differential metabolites (CFA + saline VS saline); Table S3: Up-regulated differential metabolites (CFA + 4 mg/kg LAH VS CFA + saline); Table S4: Down-regulated differential metabolites CFA+4 mg/kg LAH VS CFA + saline); Table S5: Up-regulated differential metabolites (CFA + 8 mg/kg LAH VS CFA + saline); Table S6: Down-regulated differential metabolites (CFA + 8 mg/kg LAH VS CFA + saline); Table S7: Up-regulated metabolic pathways (CFA + saline VS saline); Table S8: Down-regulated metabolic pathways (CFA + saline VS saline); Table S9: Up-regulated metabolic pathways (CFA + 4 mg/kg LAH VS CFA + saline); Table S10: Down-regulated metabolic pathways (CFA + 4 mg/kg LAH VS CFA + saline); Table S11: Up-regulated metabolic pathways (CFA +8 mg/kg LAH VS CFA + saline); Table S12: Down-regulated metabolic pathways (CFA +8 mg/kg LAH VS CFA + saline).

## Abbreviations

CFA: Complete Freund’s adjuvant; LA, lappaconitine; LAH, lappaconitine hydrobromide; DRG, dorsal root ganglion; IASP, International Pain Society; NHIS, National Health Interview Survey; iNOS, inducible nitric oxide synthase; PGE2, prostaglandin E2; PUFAs, polyunsaturated fatty acids; AA, arachidonic acids; CYP450, cytochrome P450; EETs, epoxide-eicosatrienoic acids; 9-HODE, 9-hydroxyoctadecadienoic acid;13-HODE, 13-hydroxyoctadecadienoic acid; COX, cyclooxygenase; TRPV1, transient receptor potential cation channel subfamily V member 1; EPA, eicosapentaenoic acid; ALA, alpha-linolenic acid; NPD1, neuroprotectin D1; DHA, docosahexaenoic acid; RXR, retinoid X receptor; RA, retinoic acid; PPAR, peroxisome proliferator-activated receptor; ATRA, all-trans retinoic acid; RARβ, Retinoic acid receptor beta; PPAR γ, peroxisome proliferator-activated receptor gamma; 9-cisRA, 9-cisretinoic acid. Liquid chromatography-Mass spectrometer/Mass spectrometer, LC-MS/MS; quality control, QC; Ultra-high performance liquid-chromatography, UHPLC; Mass spectrometric, MS; data-dependent acquisition, DDA; radio frequency, RF. Orthogonal Partial Least-Squares Discriminant Analysis, OPLS-DA.
