# Supplementary material for: A Metabolomic Study of the Analgesic Effect of Lappaconitine Hydrobromide (LAH) on Inflammatory Pain
Source: Metabolites. 2022 Sep 29;12(10):923. doi: 10.3390/metabo12100923 (PMC9606904; doi:10.3390/metabo12100923)
Supplement: Supplementary file 1 [file metabolites-12-00923-s001.zip › metabolites-1878192-supplementary.pdf]

Supplementary Information

A Metabolomic Study of the Analgesic Effect of Lappaconitine Hydrobromide (LAH) On inflammatory Pain

Xu Li[a,b,c] , Xueqi Wang[a], Zhengdou Li[a], Ying Mao[a], Zhao Liu[d], Xiaoxiao Liua,[e],

Xinliang Zhu[a,b,c,*], Ji Zhang[a,b,c,*]

[a] College of Life Science, Northwest Normal University, Lanzhou 730070, China

[b] Institute of New Rural Development, Northwest Normal University, Lanzhou 730070, China

[c] Bioactive Products Engineering Research Center for Gansu Distinctive Plants, Lanzhou 730070, China

[d] Department of Orthopaedic Surgery, Orthopaedic Institute, The First Affiliated Hospital, Soochow University, Suzhou 215006, Jiangsu Province, China

[e] Lanzhou Institute of Food and Drug Control, Lanzhou 740050, China

"*" Corresponding author

*Correspondence to be addressed to:

Xinliang Zhu: xzhuaf@nwnu.edu.cn

Ji Zhang： zhangj@nwnu.edu.cn

Table 1. Up-regulated differential metabolites (CFA+saline VS saline)
Table 2. Down-regulated differential metabolites (CFA+saline VS saline)
Table 3. Up-regulated differential metabolites (CFA+4mg/kg LAH VS CFA+saline)
Table 4. Down-regulated differential metabolites CFA+4mg/kg LAH VS CFA+saline)
Table 5. Up-regulated differential metabolites (CFA +8mg/kg LAH VS CFA+saline)
Table 6. Down-regulated differential metabolites (CFA+8mg/kg LAH VS CFA+saline)
Table 7. Up- regulated metabolic pathways (CFA+saline VS saline)
Table 8. Down- regulated metabolic pathways (CFA+saline VS saline)
Table 9. Up- regulated metabolic pathways (CFA+4mg/kg LAH VS CFA+saline)
Table 10. Down- regulated metabolic pathways (CFA+4mg/kg LAH VS CFA+saline)
Table 11. Up- regulated metabolic pathways (CFA +8mg/kg LAH VS CFA+saline)
Table 12. Down- regulated metabolic pathways (CFA +8mg/kg LAH VS CFA+saline)

Table S1. Up-regulated differential metabolites (CFA+saline VS saline)

m.z	FC	log2(FC)	raw. pval	VIP	Compound	Formula	HMDBID
816.5781	19.247	4.2666	0.032081	1.948541	PS (14:0/24:1(15Z))	C44H84NO10P;	HMDB0112307
818.5905	18.994	4.2475	0.019786	2.178607	PS (18:0/20:0)	C44H86NO10P;	HMDB0010164
367.1015	6.2812	2.6511	0.010204	2.072798	3-Feruloylquinic acid	C17H20O9;	HMDB0030669
212.0741	4.9261	2.3005	0.036462	1.753544	2-(4-Methyl-5-thiazolyl)ethyl butanoate	C10H15NO2S;	HMDB0032418
407.2792	4.4665	2.1591	0.000146	1.224039	3alpha,7alpha,12beta-Trihydroxy-5beta-cholanoic acid	C24H40O5;	HMDB0000312
310.1777	3.3937	1.7628	0.002609	2.446785	Lysyltyrosine	C15H23N3O4;	HMDB0028963
242.1759	3.2313	1.6921	0.019397	1.979134	N-Undecanoylglycine	C13H25NO3;	HMDB0013286
192.0167	2.9029	1.5375	0.005277	1.795592	Phosphocreatinine	C4H8N3O4P;	HMDB0041624
226.0345	2.7935	1.4821	0.017675	1.524057	5-(2'-Carboxyethyl)-4,6-Dihydroxypicolinate	C9H9NO6;	HMDB0006794
336.1291	2.7309	1.4494	0.025126	1.251445	4-O-alpha-D-Galactopyranosylcalystegine B2	C13H23NO9;	HMDB0038228
181.9905	2.5063	1.3255	0.048707	1.854902	Saccharin	C7H5NO3S;	HMDB0029723
216.1232	2.4684	1.3036	0.004883	1.175059	Propionylcarnitine	C10H19NO4;	HMDB0000824
294.1829	2.2953	1.1987	0.01333	2.408093	Lysylphenylalanine	C15H23N3O3;	HMDB0028958
352.132	2.2358	1.1608	2.79E-06	2.319153	Adinazolam	C19H18ClN5;	HMDB0014686
207.0137	2.0535	1.0381	0.046342	1.553307	Garcinia acid	C6H8O8;	HMDB0031159
333.2447	9.4604	3.2419	0.036185	1.979704	16-a-Hydroxypregnenolone	C21H32O3;	HMDB0000315
301.2147	6.009	2.5871	0.000105	1.514839	all-trans-Retinoic acid	C20H28O2;	HMDB0001852
508.0007	4.2454	2.0859	0.016462	2.127086	Adenosine triphosphate	C10H16N5O13P3;	HMDB0000538
420.3082	4.1925	2.0678	0.002262	2.45446	Stearidonyl carnitine	C25H41NO4;	HMDB0006463
170.0593	3.9702	1.9892	0.013284	1.67924	L-Isoleucine	C6H13NO2;	HMDB0000172
760.5817	3.9234	1.9721	0.026488	2.476718	PC (14:0/20:1(11Z))	C42H82NO8P;	HMDB0007879
138.0658	3.5914	1.8445	0.004168	1.660407	Isoniazid	C6H7N3O;	HMDB0015086
258.1087	2.4977	1.3206	0.036986	1.712688	5-Methylcytidine	C10H15N3O5;	HMDB0000982
338.3398	2.4043	1.2656	0.044635	1.687575	Arachidyl alcohol	C20H42O;	HMDB0011619
159.0911	2.2995	1.2013	0.013214	2.168322	L-Tryptophan	C11H12N2O2;	HMDB0000929
118.086	2.1257	1.0879	0.049708	1.857006	L-Valine	C5H11NO2;	HMDB0000883
126.0659	2.1058	1.0744	0.043581	1.467167	5-Methylcytidine	C10H15N3O5;	HMDB0000982
276.1255	2.0624	1.0443	0.008324	2.042136	(±)-Ribaline	C15H17NO4;	HMDB0033345

Table S2. Down-regulated differential metabolites (CFA+saline VS saline)

m.z	FC	log2(FC)	raw. pval	VIP	Compound	Formula	HMDBID
143.0391	0.45822	-1.1259	0.04157	1.952116	2-Propionyl-2-thiazoline	C6H9NOS;	HMDB0032493
286.164	0.4498	-1.1526	0.012345	1.938468	N-Monodesmethyl-rizatriptan	C15H19N5O;	HMDB0060847
167.0698	0.43542	-1.1995	0.012549	2.068671	3-(3-Hydroxyphenyl) propanoic acid	C9H10O3;	HMDB0000375
454.1693	0.42732	-1.2266	0.022614	1.381900	O-Methyl-4-[(2',3',4'-tri-O-acetyl-alpha-L-rhamnosyloxy) benzyl]carbamate	C21H27NO10;	HMDB0031948
261.1089	0.36493	-1.4543	0.042592	1.639336	Glutamylhydroxyproline	C10H16N2O6;	HMDB0011161
175.1111	0.36226	-1.4649	0.007902	2.193182	6-Phenyl-3-hexen-2-one	C12H14O;	HMDB0031622
526.2909	0.32139	-1.6376	0.029307	1.727389	LysoPE (0:0/22:6(4Z,7Z,10Z,13Z,16Z,19Z))	C27H44NO7P;	HMDB0011496
317.0698	0.30847	-1.6968	0.00612	2.437929	4'-Hydroxyclobazam	C16H13ClN2O3;	HMDB0060771
214.0153	0.2913	-1.7794	0.028978	1.674660	Carmustine	C5H9Cl2N3O2;	HMDB0014407
365.1340	0.27546	-1.8601	0.008734	1.942993	2-(acetylamino)-1,5-anhydro-2-deoxy-3-O-b-D-galactopyranosyl-D-arabino-Hex-1-enitol	C14H23NO10;	HMDB0002278
129.0197	0.2674	-1.9029	0.015293	1.843744	5-Hydroxy-2-furoic acid	C5H4O4;	HMDB0059784
178.1219	0.21008	-2.251	0.011883	1.721154	2-Pentylfuran	C9H14O;	HMDB0013824
119.0853	0.06740	-3.8909	0.002497	2.083025	Longistylin C	C20H22O;	HMDB0038544
276.1432	0.05848	-4.0958	5.96E-11	2.689502	Glutarylcarnitine	C12H21NO6;	HMDB0013130
407.1644	0.05801	-4.1074	5.97E-05	2.458843	3-(3-hydroxyphenyl)-2-phenyl-4-[(E)-2-phenylethenyl]-2,3-dihydro-1-benzofuran-6-ol	C28H22O3;	HMDB0129161
343.0520	0.04345	-4.5244	0.021633	1.631803	N-Acetylaspartylglutamic acid	C11H16N2O8;	HMDB0001067
323.0754	0.41036	-1.285	0.033734	1.623938	Mahaleboside	C15H16O8;	HMDB0032984
435.2515	0.37242	-1.425	0.009463	1.867405	LysoPA (0:0/18:1(9Z))	C21H41O7P;	HMDB0007851
513.6644	0.37098	-1.4306	0.046733	1.846804	Linoleoyl-CoA	C39H66N7O17P3S;	HMDB0001064
311.1683	0.28464	-1.8128	0.008068	2.065756	N-Undecylbenzenesulfonic acid	C17H28O3S;	HMDB0032549
420.9711	0.24263	-2.0432	0.010562	1.665603	Chlorophenol red	C19H12Cl2O5S;	HMDB0036699
524.2998	0.23544	-2.0865	0.019572	1.958424	1-Stearoylglycerophosphoserine	C24H48NO9P;	HMDB0061698
886.5536	0.22221	-2.17	0.031776	1.865563	PI (16:0/22:4(10Z,13Z,16Z,19Z))	C47H83O13P;	HMDB0009793
889.5796	0.14806	-2.7557	0.008072	1.189033	PI (16:0/22:3(10Z,13Z,16Z))	C47H85O13P;	HMDB0009792
325.1841	0.081417	-3.6185	0.00119	2.415962	2-Dodecylbenzenesulfonic acid	C18H30O3S;	HMDB0031031

Table S3. Up-regulated differential metabolites (CFA + 4 mg/kg LAH VS CFA+saline)

m.z	FC	log2(FC)	raw. pval	vip	Compound	Formula	HMDBID
343.0520	38.026	5.2489	0.008051	1.999891	N-Acetylaspartylglutamic acid	C11H16N2O8;	HMDB0001067
113.0344	10.81	3.4343	0.036502	1.57608	Uracil	C4H4N2O2;	HMDB0000300
342.2624	10.101	3.3364	0.005518	1.094027	trans-2-Dodecenoylcarnitine	C19H35NO4;	HMDB0013326
311.2568	6.0639	2.6002	0.012234	1.046699	(R)-2-Hydroxysterculic acid	C19H34O3;	HMDB0031060
167.0919	5.9836	2.581	0.002561	1.841763	1-Deoxy-D-glucitol	C6H14O5;	HMDB0041500
149.0818	5.5323	2.4679	0.010205	1.864692	Mevalonic acid	C6H12O4;	HMDB0000227
175.1111	4.478	2.1629	0.000704	2.33527	6-Phenyl-3-hexen-2-one	C12H14O;	HMDB0031622
218.1379	4.1485	2.0526	2.79E-05	2.315508	Propionylcarnitine	C10H19NO4;	HMDB0000824
165.0763	4.0661	2.0236	0.01614	1.794793	L-Fucose	C6H12O5;	HMDB0000174
422.3241	3.9481	1.9811	0.013104	1.767094	Gamma-linolenyl carnitine	C25H43NO4;	HMDB0006318
215.0694	3.8021	1.9268	0.027243	1.611336	Phenyl salicylate	C13H10O3;	HMDB0032018
288.1975	3.4547	1.7886	0.002154	1.880929	N-[2-(4-Prenyloxyphenyl)ethyl]tiglamide	C18H25NO2;	HMDB0041142
276.1950	3.2512	1.701	0.00489	1.462375	Citronellyl anthranilate	C17H25NO2;	HMDB0032208
135.0660	3.0067	1.5882	0.046777	1.192951	2,3-Dihydroxyvaleric acid	C5H10O4;	HMDB0000421
111.0441	2.6123	1.3853	0.000961	2.24718	Pyrocatechol	C6H6O2;	HMDB0000957
281.0497	2.596	1.3763	0.006069	1.468420	6-[(2-carboxyacetyl)oxy]-3,4,5-trihydroxyoxane-2-carboxylic acid	C9H12O10;	HMDB0127482
283.0464	2.5863	1.3709	0.012716	1.613437	Bikojic acid	C12H10O8;	HMDB0034034
118.0861	2.5266	1.3372	0.023214	1.604875	L-Valine	C5H11NO2;	HMDB0000883
143.0391	2.5187	1.3327	0.014033	1.930327	2-Propionyl-2-thiazoline	C6H9NOS;	HMDB0032493
179.0920	2.401	1.2636	0.019626	1.335832	Dibutyl disulfide	C8H18S2;	HMDB0029569
112.0505	2.2556	1.1735	0.025012	1.53286	Cytosine	C4H5N3O;	HMDB0000630
149.0817	2.1018	1.0716	0.046016	1.480737	Mevalonic acid	C6H12O4;	HMDB0000227
227.2011	45.255	5.5	0.045449	2.113848	Myristic acid	C14H28O2;	HMDB0000806
187.0060	4.1783	2.0629	0.046469	1.374291	p-Cresol sulfate	C7H8O4S;	HMDB0011635
126.0016	3.6637	1.8733	0.046044	1.941178	2-Acetylthiazole	C5H5NOS;	HMDB0032964
311.1683	3.1723	1.6655	0.020282	2.455782	N-Undecylbenzenesulfonic acid	C17H28O3S;	HMDB0032549
833.5196	3.1519	1.6562	0.005256	1.236311	PI (16:0/18:2(9Z,12Z))	C43H79O13P;	HMDB0009784
277.2172	2.706	1.4361	0.011989	2.517848	alpha-Linolenic acid	C18H30O2;	HMDB0001388
913.5774	2.5808	1.3678	0.00342	1.457307	PI (18:0/22:4(10Z,13Z,16Z,19Z))	C49H87O13P;	HMDB0009817

Table S4. Down-regulated differential metabolites (CFA + 4 mg/kg LAH VS CFA+saline)

m.z	FC	log2(FC)	raw.pval	vip	Compound	Formula	HMDBID
244.1531	0.42253	-1.2429	0.005747	1.134147	Tiglylcarnitine	C12H21NO4;	HMDB0002366
357.2772	0.39972	-1.3229	0.046704	1.088948	Tetracosahexaenoic acid	C24H36O2;	HMDB0002007
302.1741	0.39359	-1.3452	0.016739	1.116391	Dobutamine	C18H23NO3;	HMDB0014979
407.1644	0.31026	-1.6885	0.002642	2.048554	3-(3-hydroxyphenyl)-2-phenyl-4-[(E)-2-phenylethenyl]-2,3-dihydro-1-benzofuran-6-ol	C28H22O3;	HMDB0129161
242.1531	0.27707	-1.8517	0.00011	2.545078	2-Carboxy-4-dodecanolide	C13H22O4;	HMDB0030987
295.1717	0.23224	-2.1063	0.030125	2.074916	(E, E)-1,6-bis(4-methoxyphenyl)-1,5-hexadiene	C20H22O2;	HMDB0035443
198.0868	0.16581	-2.5924	0.001023	2.036503	N-Acetylhistidine	C8H11N3O3;	HMDB0032055
325.1222	0.16346	-2.613	0.02373	1.555477	Acetohexamide	C15H20N2O4S;	HMDB0014558
301.2147	0.12171	-3.0384	0.000942	1.543497	all-trans-Retinoic acid	C20H28O2;	HMDB0001852
276.1432	0.11464	-3.1248	1.21E-05	2.410398	Glutarylcarnitine	C12H21NO6;	HMDB0013130
241.1535	0.1054	-3.2461	0.005473	1.998421	Pirbuterol	C12H20N2O3;	HMDB0015407
182.1169	0.094529	-3.4031	0.006765	2.235141	beta-O-Methylynephrine	C10H15NO2;	HMDB0033868
334.3083	0.091549	-3.4493	0.016727	1.885209	2,4,12-Octadecatrienoic acid isobutylamide	C22H39NO;	HMDB0032033
276.1255	0.088616	-3.4963	0.02117	1.691246	(±)-Ribaline	C15H17NO4;	HMDB0033345
133.1006	0.087262	-3.5185	0.028946	1.574617	p-Mentha-1,3,5,8-tetraene	C10H12;	HMDB0029641
273.147	0.046742	-4.4191	0.020392	2.18364	(S)-Verimol F	C17H20O3;	HMDB0036558
162.0756	0.003337	-8.2273	0.01967	1.757042	L-Proline	C5H9NO2;	HMDB0000162
310.1777	0.47222	-1.0825	0.046027	2.822441	Lysyltyrosine	C15H23N3O4;	HMDB0028963
349.2383	0.4437	-1.1723	0.001167	1.043016	Tetrahydrocorticosterone	C21H34O4;	HMDB0000268
612.1476	0.40157	-1.3163	0.002397	2.472912	Delphinidin 3-(3''-p-coumaroylglucoside)	C30H27O14;	HMDB0030099
242.1759	0.33315	-1.5857	0.048106	2.237574	N-Undecanoylglycine	C13H25NO3;	HMDB0013286
407.2792	0.20467	-2.2886	4.78E-07	1.883707	3alpha,7alpha,12beta-Trihydroxy-5beta-cholanoic acid	C24H40O5;	HMDB0000312
225.0877	0.006665	-7.2291	0.010084	2.365000	Porphobilinogen	C10H14N2O4;	HMDB0000245

Table S5. Up-regulated differential metabolites (CFA + 8 mg/kg LAH VS CFA+saline)

m.z	FC	log2(FC)	raw.pval	VIP	Compound	Formula	HMDBID
342.2624	10.467	3.3878	0.015358	2.240215	trans-2-Dodecenoylcarnitine	C19H35NO4;	HMDB0013326
188.0698	5.141	2.362	0.016022	2.054379	Indoleacrylic acid	C11H9NO2	HMDB0000734
277.179	4.099	2.0353	0.000674	2.190074	Cyclandelate	C17H24O3;	HMDB0015586
422.3241	3.9896	1.9962	0.007153	2.409213	Gamma-linolenyl carnitine	C25H43NO4;	HMDB0006318
149.0818	3.5442	1.8255	0.048059	1.860642	Mevalonic acid	C6H12O4;	HMDB0000227
218.1379	2.5449	1.3476	0.030549	2.19103	Propionylcarnitine	C10H19NO4;	HMDB0000824
279.1768	2.4546	1.2955	0.004172	1.919588	Longistylin C	C20H22O;	HMDB0038544
288.1975	2.4412	1.2876	6.95E-05	1.712916	N-[2-(4-Prenyloxyphenyl)ethyl]tiglamide	C18H25NO2;	HMDB0041142
295.1717	2.4329	1.2827	0.001895	2.157766	(E, E)-1,6-bis(4-methoxyphenyl)-1,5-hexadiene	C20H22O2;	HMDB0035443
369.2809	2.3578	1.2375	0.002386	2.165357	(R)-3,4-Dihydro-2-(4,8,12-trimethyl-3,7,11-tridecatrienyl)-2H-1-benzopyran-6-ol	C25H36O2;	HMDB0036013
241.2162	94.692	6.5652	0.030731	1.844465	Pentadecanoic acid	C15H30O2;	HMDB0000826
253.2169	45.488	5.5074	0.013399	2.237681	Hypogeic acid	C16H30O2;	HMDB0002186
239.0772	26.814	4.7449	0.029605	1.894992	D-glycero-L-galacto-Octulose	C8H16O8;	HMDB0029954
889.5796	21.022	4.3938	0.00046	1.590157	PI (16:0/22:3(10Z,13Z,16Z))	C47H85O13P;	HMDB0009792
325.1841	9.1557	3.1947	0.016962	2.205195	2-Dodecylbenzenesulfonic acid	C18H30O3S;	HMDB0031031
524.2998	7.4001	2.8875	0.002134	1.410184	1-Stearoylglycerophosphoserine	C24H48NO9P;	HMDB0061698
888.5734	6.2957	2.6544	0.000165	1.820788	PI (16:0/22:3(10Z,13Z,16Z))	C47H85O13P;	HMDB0009792
269.2483	5.7606	2.5262	0.000602	2.688105	Heptadecanoic acid	C17H34O2;	HMDB0002259
239.0719	4.8937	2.2909	0.018057	1.926519	2',5'-Dihydroxychalcone	C15H12O3;	HMDB0032129
277.2172	4.4012	2.1379	0.02179	2.074078	alpha-Linolenic acid	C18H30O2;	HMDB0001388
863.5634	3.3454	1.7422	0.000794	1.499883	PI (18:0/18:1(9Z))	C45H85O13P;	HMDB0240667
297.2428	2.0994	1.07	0.021124	1.637517	3-Oxooctadecanoic acid	C18H34O3;	HMDB0010736

Table S6. Down-regulated differential metabolites (CFA + 8 mg/kg LAH VS CFA+saline)

m.z	FC	log2(FC)	raw.pval	VIP	Compound	Formula	HMDBID
310.1777	0.44383	-1.1719	0.004815	2.512193	Lysyltyrosine	C15H23N3O4;	HMDB0028963
166.9738	0.42296	-1.2414	0.016765	1.644247	Phosphoenolpyruvic acid	C3H5O6P;	HMDB0000263
407.2792	0.41087	-1.2832	0.003796	1.722873	3alpha,7alpha,12beta-Trihydroxy-5beta-cholanoic acid	C24H40O5;	HMDB0000312
182.0458	0.33742	-1.5674	0.008578	2.250291	4-Pyridoxic acid	C8H9NO4;	HMDB0000017
352.132	0.33282	-1.5872	0.013361	2.127306	Adinazolam	C19H18ClN5;	HMDB0014686
373.0981	0.25001	-2	0.016015	2.0525	Succinylaminoimidazole carboxamide riboside	C13H18N4O9;	HMDB0240295
335.2931	0.49848	-1.0044	0.016054	1.447006	Docosatrienoic acid	C22H38O2;	HMDB0002823
338.3398	0.44543	-1.1667	0.048262	1.690396	Arachidyl alcohol	C20H42O;	HMDB0011619
302.1741	0.40592	-1.3007	0.012193	2.225286	Dobutamine	C18H23NO3;	HMDB0014979
355.2618	0.37155	-1.4284	0.008778	2.004698	1,1'-[1,12-Dodecanediylbis(oxy)]bisbenzene	C24H34O2;	HMDB0039760
333.2447	0.32921	-1.6029	0.003163	1.046064	16-a-Hydroxypregnenolone	C21H32O3;	HMDB0000315
276.1432	0.32676	-1.6137	0.008224	1.900745	Glutarylcarnitine	C12H21NO6;	HMDB0013130
407.1644	0.25316	-1.9819	0.001313	2.143949	3-(3-hydroxyphenyl)-2-phenyl-4-[(E)-2-phenylethenyl]-2,3-dihydro-1-benzofuran-6-ol	C28H22O3;	HMDB0129161
666.1289	0.22873	-2.1283	0.009734	2.219455	NADH	C21H29N7O14P2;	HMDB0001487
276.1255	0.21511	-2.2169	0.000349	2.271959	(±)-Ribaline	C15H17NO4;	HMDB0033345
198.0868	0.20791	-2.266	0.00213	1.883446	N-Acetylhistidine	C8H11N3O3;	HMDB0032055
114.0911	0.18003	-2.4737	0.005872	2.209449	2,5-Dihydro-2,4,5-trimethyloxazole	C6H11NO;	HMDB0031199
301.2147	0.15967	-2.6469	0.000454	2.359639	all-trans-Retinoic acid	C20H28O2;	HMDB0001852
425.3431	0.15493	-2.6903	0.001518	1.108938	alpha-Tocotrienol	C29H44O2;	HMDB0006327
182.1169	0.14407	-2.7952	0.036291	2.380631	beta-O-Methylynephrine	C10H15NO2;	HMDB0033868
330.0712	0.12053	-3.0526	0.000645	2.199608	Glutathione	C10H17N3O6S	HMDB0000125
303.1357	0.095353	-3.3906	0.018477	2.294517	Arginylglutamine	C11H22N6O4	HMDB0028707
480.3069	0.055113	-4.1815	0.031055	2.125346	LysoPE(0:0/18:1(11Z))	C23H46NO7P;	HMDB0011475
297.1878	0.022926	-5.4469	1.62E-05	2.543366	17a-Ethynylestradiol	C20H24O2;	HMDB0001926
273.147	0.010241	-6.6095	0.011057	2.339072	(S)-Verimol F	C17H20O3;	HMDB0036558

Table S7. Up- regulated metabolic pathways (CFA+saline VS saline)

Pathway	Total	Expected	Hits	Raw p	#NAME?	Holm adjust	FDR	Impact
Valine, leucine and isoleucine biosynthesis	8	0.074221	2	0.002169	2.6637	0.1822	0.1822	0
Aminoacyl-tRNA biosynthesis	48	0.44533	3	0.008596	2.0657	0.71345	0.36102	0
Glycerophospholipid metabolism	36	0.334	2	0.04207	1.376	1	0.85675	0.14147
Linoleic acid metabolism	5	0.046388	1	0.045595	1.3411	1	0.85675	0
Valine, leucine and isoleucine degradation	40	0.37111	2	0.050997	1.2925	1	0.85675	0
alpha-Linolenic acid metabolism	13	0.12061	1	0.11455	0.941	1	1	0
Retinol metabolism	16	0.14844	1	0.13919	0.85638	1	1	0.22754
Pantothenate and CoA biosynthesis	19	0.17628	1	0.1632	0.78728	1	1	0
Arachidonic acid metabolism	36	0.334	1	0.28789	0.54077	1	1	0
Tryptophan metabolism	41	0.38038	1	0.32114	0.4933	1	1	0.14305
Purine metabolism	66	0.61233	1	0.46682	0.33085	1	1	0.00702

Table S8. Down- regulated metabolic pathways (CFA+saline VS saline)

Pathway	Total	Expected	Hits	Raw p	#NAME?	Holm adjust	FDR	Impact
Glycerolipid metabolism	16	0.074221	1	0.072041	1.1424	1	1	0.01246
Phosphatidylinositol signaling system	28	0.12989	1	0.12311	0.90971	1	1	0.00152
Alanine, aspartate and glutamate metabolism	28	0.12989	1	0.12311	0.90971	1	1	0.04808
Biosynthesis of unsaturated fatty acids	36	0.167	1	0.1558	0.80744	1	1	0.09091
Glycerophospholipid metabolism	36	0.167	1	0.1558	0.80744	1	1	0.13987

Table S9. Up- regulated metabolic pathways (CFA + 4 mg/kg LAH VS CFA+saline)

Pathway	Total	Expected	Hits	Raw p	#NAME?	Holm adjust	FDR	Impact
Pantothenate and CoA biosynthesis	19	0.16368	2	0.010791	1.9669	0.90642	0.90642	0
Valine, leucine and isoleucine biosynthesis	8	0.06892	1	0.067028	1.1737	1	1	0
alpha-Linolenic acid metabolism	13	0.11199	1	0.10679	0.97148	1	1	0.33333
Fructose and mannose metabolism	18	0.15507	1	0.14498	0.83869	1	1	0
Terpenoid backbone biosynthesis	18	0.15507	1	0.14498	0.83869	1	1	0.11429
beta-Alanine metabolism	21	0.18091	1	0.16716	0.77686	1	1	0
Alanine, aspartate and glutamate metabolism	28	0.24122	1	0.21688	0.66378	1	1	0.04808
Biosynthesis of unsaturated fatty acids	36	0.31014	1	0.27033	0.5681	1	1	0
Amino sugar and nucleotide sugar metabolism	37	0.31875	1	0.27677	0.55787	1	1	0
Pyrimidine metabolism	39	0.33598	1	0.2895	0.53836	1	1	0.0743
Valine, leucine and isoleucine degradation	40	0.3446	1	0.29578	0.52903	1	1	0
Tryptophan metabolism	41	0.35321	1	0.30201	0.51998	1	1	0.00202
Fatty acid biosynthesis	47	0.4049	1	0.33835	0.47064	1	1	0
Aminoacyl-tRNA biosynthesis	48	0.41352	1	0.34423	0.46315	1	1	0

Table S10. Down- regulated metabolic pathways (CFA + 4 mg/kg LAH VS CFA+saline)

Pathway	Total	Expected	Hits	Raw p	#NAME?	Holm adjust	FDR	Impact
Retinol metabolism	16	0.095427	1	0.091712	1.0376	1	1	0.22754
Porphyrin and chlorophyll metabolism	30	0.17893	1	0.16574	0.78056	1	1	0.05288
Arginine and proline metabolism	38	0.22664	1	0.20559	0.68699	1	1	0.0778
Aminoacyl-tRNA biosynthesis	48	0.28628	1	0.25302	0.59684	1	1	0
Steroid hormone biosynthesis	77	0.45924	1	0.37666	0.42405	1	1	0

Table S11. Up- regulated metabolic pathways (CFA + 8 mg/kg LAH VS CFA+saline)

Pathway	Total	Expected	Hits	Raw p	#NAME?	Holm adjust	FDR	Impact
alpha-Linolenic acid metabolism	13	0.043075	1	0.042394	1.3727	1	1	0.33333
Terpenoid backbone biosynthesis	18	0.059642	1	0.058312	1.2342	1	1	0.11429
Biosynthesis of unsaturated fatty acids	36	0.11928	1	0.11387	0.94359	1	1	0

Table S12. Down- regulated metabolic pathways (CFA + 8 mg/kg LAH VS CFA+saline)

Pathway	Total	Expected	Hits	Raw p	#NAME?	Holm adjust	FDR	Impact
Vitamin B6 metabolism	9	0.059642	1	0.058236	1.2348	1	1	0
Retinol metabolism	16	0.10603	1	0.1014	0.99396	1	1	0.22754
Citrate cycle (TCA cycle)	20	0.13254	1	0.12526	0.90219	1	1	0
Pyruvate metabolism	22	0.14579	1	0.13697	0.86337	1	1	0
Glycolysis / Gluconeogenesis	26	0.1723	1	0.15998	0.79594	1	1	0.1055
